# Automated vacuum drying kinetics, thermodynamics, and economic analysis of sage leaves

**DOI:** 10.1038/s41598-025-03367-z

**Published:** 2025-05-29

**Authors:** Nabil Eldesokey Mansour, Khaled A. Metwally, Aml Abubakr Tantawy, Ahmed Elbeltagi, Ali Salem, Ahmed Z. Dewidar, Abdelaziz M. Okasha, Moustapha Eid Moustapha, Abdallah Elshawadfy Elwakeel

**Affiliations:** 1 Agricultural Engineering Department, Faculty of Agriculture, Damanhour University, Damanhour, Egypt; 2 Soil and Water Sciences Department, Faculty of Technology and Development, Zagazig University, Zagazig, 44519 Egypt; 3 Food Science Department, Faculty of Agriculture, Beni-Suef University, Beni-Suef, 65211 Egypt; 4 Agricultural Engineering Department, Faculty of Agriculture, Mansoura University, Mansoura, 35516 Egypt; 5 Civil Engineering Department, Faculty of Engineering, Minia University, Minia 61111, Egypt; 6 Structural Diagnostics and Analysis Research Group, Faculty of Engineering and Information Technology, University of Pécs, Pécs 7622, Hungary; 7 Prince Sultan Bin Abdulaziz International Prize for Water Chair, Prince Sultan Institute for Environmental, Water and Desert Research, King Saud University, Riyadh, 11451 Saudi Arabia; 8 Department of Agricultural Engineering, College of Food and Agriculture Sciences, King Saud University, Riyadh, 11451 Saudi Arabia; 9 Department of Agricultural Engineering, Faculty of Agriculture, Kafrelsheikh University, Kafr El-Sheikh, 33516 Egypt; 10 Department of Chemistry, College of Science and Humanities, Prince Sattam bin Abdulaziz University, Al-Kharj, 11942 Saudi Arabia; 11 Agricultural Engineering Department, Faculty of Agriculture and Natural Resources, Aswan University, Aswan, 81528 Egypt

**Keywords:** Activation entropy, Free energy of Gibbs, Internet of things (IoT), Medicinal and aromatic plants, Thin layer modeling, Plant sciences, Energy science and technology

## Abstract

Vacuum drying of sage leaves is important for preserving their essential oils, flavor, and medicinal properties by reducing oxidation and thermal degradation, but previous research has not investigated its impact on drying speed, thermodynamic properties, mathematical modeling, or economic viability. This study employed an automatic vacuum dryer at temperatures of 40 °C, 50 °C, and 60 °C under different pressure conditions (atmospheric, -5 kPa, and − 10 kPa) with a 1 cm layer thickness. Results showed that increasing temperature and decreasing pressure significantly improved drying efficiency, reducing the process time to just 90 min while achieving a drying rate of 22.34 kg water/kg dry matter/h and an effective moisture diffusivity of 6.716 × 10⁻⁹ m²/s under optimal conditions (60 °C and − 10 kPa). The Page model was identified as the most suitable for describing the thin-layer drying behavior. Thermodynamic analysis revealed activation energy values between 19.4 and 37.7 kJ/mol, with activation enthalpy decreasing at higher temperatures and lower pressures. The negative activation entropy values indicated chemical adsorption or structural modifications during drying. From an economic perspective, the most efficient drying conditions reduced the payback period to less than two months, demonstrating strong commercial potential. These findings highlight the industrial promise of vacuum drying for herb processing, with future research opportunities in process optimization, application to other herbs, and sustainability assessments to further enhance efficiency and economic benefits.

## Introduction

Sage (*Salvia officinalis L*.), a versatile and aromatic herb, is globally valued for its culinary, medicinal, and ornamental uses^1,2^. Originating from the Mediterranean region^3^, sage is renowned for its distinctive flavor and health benefits, including anti-inflammatory, antioxidant, and antimicrobial properties. Its essential oils and bioactive compounds, such as thujone and rosmarinic acid, make it a key ingredient in herbal remedies, cosmetics, and natural health products^4–6^. Globally, sage is cultivated in temperate climates with well-drained soils, with major producers including countries in Europe, North America, and the Middle East^7,8^.

Sage production is gaining importance in Libya due to the country’s favorable climate and agricultural expertise. Where grown primarily in regions with adequate sunlight and irrigation, sage is prized for its high quality and potency. Local farmers cultivate sage for both domestic use and export, contributing to the country’s agricultural economy. The herb is processed into dried leaves, teas, and essential oils, catering to both local markets and international demand. Libya’s sage production not only supports sustainable farming practices but also highlights the country’s potential as a key player in the global herbal industry^7,9–11^.

Drying is an essential process for preserving the quality, flavor, and medicinal properties of sage, but traditional drying methods often fall short in maintaining the herb’s integrity^12–14^. Industrial dryers, while efficient and capable of handling large volumes, typically operate at high temperatures, which can degrade heat-sensitive bioactive compounds such as essential oils, flavonoids, and phenolic acids. This thermal degradation not only diminishes sage’s aromatic and therapeutic qualities but also reduces its market value^15,16,13^. Additionally, industrial dryers are energy-intensive, contributing to higher production costs and environmental impact^17–19^. Furthermore, open sun drying, a widely used method due to its low cost and simplicity, presents its own set of challenges^20–22^. This method is highly dependent on weather conditions, making it inconsistent and time-consuming^23,24^. Moreover, sage dried in open sunlight is exposed to contamination from dust, insects, and microorganisms, which can compromise its safety and quality^25–27^. Prolonged exposure to UV radiation and fluctuating temperatures can also lead to the loss of volatile compounds and discoloration, further reducing the herb’s appeal^28,29^.

In contrast, vacuum drying has emerged as a superior alternative, addressing the limitations of traditional methods. By operating at low temperatures and in an oxygen-free environment, vacuum drying minimizes thermal degradation and oxidation, preserving sage’s essential oils, vibrant color, and bioactive compounds^30–32^. This method ensures a higher-quality product with enhanced shelf life, making it ideal for culinary, medicinal, and cosmetic applications^33,34^. Furthermore, vacuum drying is energy-efficient and scalable, offering a sustainable solution for modern herb processing. Its ability to maintain the functional and sensory properties of sage underscores its importance in the post-harvest industry, paving the way for improved product quality and economic value^35,36^.

Many previous studies were conducted on drying of sage leaves including, Venskutonis^37^ studied the effect of drying on the volatile constituents; Sellami et al.^38^ studied the effect of drying on chemical composition, and radical scavenging activity of the essential oil; Sadowska et al.^39^ evaluated effects of drying method of the concentration of compounds on sage; Esturk^40^ investigated the effect of intermittent and continuous microwave-convective air-drying characteristics of sage leaves; Hamrouni-Sellami et al.^41^ determined the effects of seven drying methods on total phenolics, flavonoids, individual phenolics, and antioxidant activity of the methanol extract of sage; Hassanain^42^ used different types of passive solar dryer for drying sage; Esturk et al.^43^ dried sage inflorescences by intermittent and continuous microwave-convective air combination; Belghit et al.^44^ experimentally studied the drying kinetics of sage in a drying tunnel working in forced convection; Doymaz et al.^45^ studied the effect of air temperature on drying kinetics, color changes and total phenolic content of sage leaves; Salehi et al.^46^ studied the effect of drying methods on rheological and textural properties, and color changes of wild sage seed gum; Şahin-Nadeem et al.^47^ experimentally studied the influence of inlet air temperature and carrier material on the production of instant soluble sage by spray drying; Baltacı et al.^48^ studied the effects of spray and freeze-drying methods on aroma compounds, sensory characteristics, physicochemical composition, antioxidant and antimicrobial properties of instant sage tea; and Amini et al.^49^ studied the effect of infrared drying on drying kinetics and color changes of wild sage seed mucilage. To date, there has been a lack of research investigating the impact of evacuated drying on sage leaves with respect to drying kinetics, thermodynamic properties, mathematical modeling, and the economic analysis of the drying process. This absence of investigation highlights a significant gap in the existing literature, emphasizing the necessity for comprehensive studies that thoroughly explore these variables. A deeper understanding of the relationship between drying methods and their economic consequences could facilitate the development of more efficient and sustainable practices within various agricultural sectors.

This research paper aims to investigate the drying kinetics of sage leaves, focusing on various parameters, including accumulated weight loss, moisture content, moisture ratio, drying rate, EMD, and activation energy. The study will also address mathematical modeling, thermodynamic properties, and economic analysis of a developed automatic vacuum dryer (DAVD) designed for sage leaves. The objective of this research is to provide critical data that will aid in identifying the optimal drying model to enhance the commercial drying process of sage leaves. The findings of this study will be of particular significance to professionals in postharvest technology who are seeking to improve the efficiency of their drying methods. Moreover, the results may assist in the formulation of industry standards that promote consistent quality in the final product. By optimizing the drying process, producers can increase their yields and enhance the marketability of sage leaves.

## Materials and methods

### Experimental setup

During the current study, sage plants were harvested from a local farm in Al-Faydiyah, Al-Jabal Al-Akhdar, Libya, and stored in a refrigerator at 4 °C before use. All drying experiments were conducted in the laboratory of the Department of Agricultural Engineering, Faculty of Agriculture, Omar Al-Mukhtar University, Libya, after the plucking season of 2021. The plants were washed with tap water to remove dirt and dust. Then they were gently watered and wiggled to dry from the water. Excess water was then wiped off with paper towels. The initial moisture content was then determined by the hot air furnace method using a laboratory furnace. An electronic balance was used to weigh the samples, with each sample weighing 100 g. The experiment was conducted in triplicate, and the average was taken. The initial moisture content before drying was 85.5%. All drying experiments were conducted with a layer thickness of 1 cm. Additionally, the sage leaf samples were dried at three operating pressures (P1 = normal atmospheric pressure (atm), P2 = -5 kPa, and P3 = -10 kPa) and three drying temperatures (T1 = 40, T2 = 50, and T3 = 60 °C). All drying experiments were conducted until constant weight and reaching the equilibrium moisture content. The moisture content during the drying process was estimated by weighing the samples periodically with load sensors every 15 min. Furthermore, the relative humidity and temperature of the air inside the dryer were measured with the developed measuring unit.

### Description of the DAVD

**Fig. 1:**
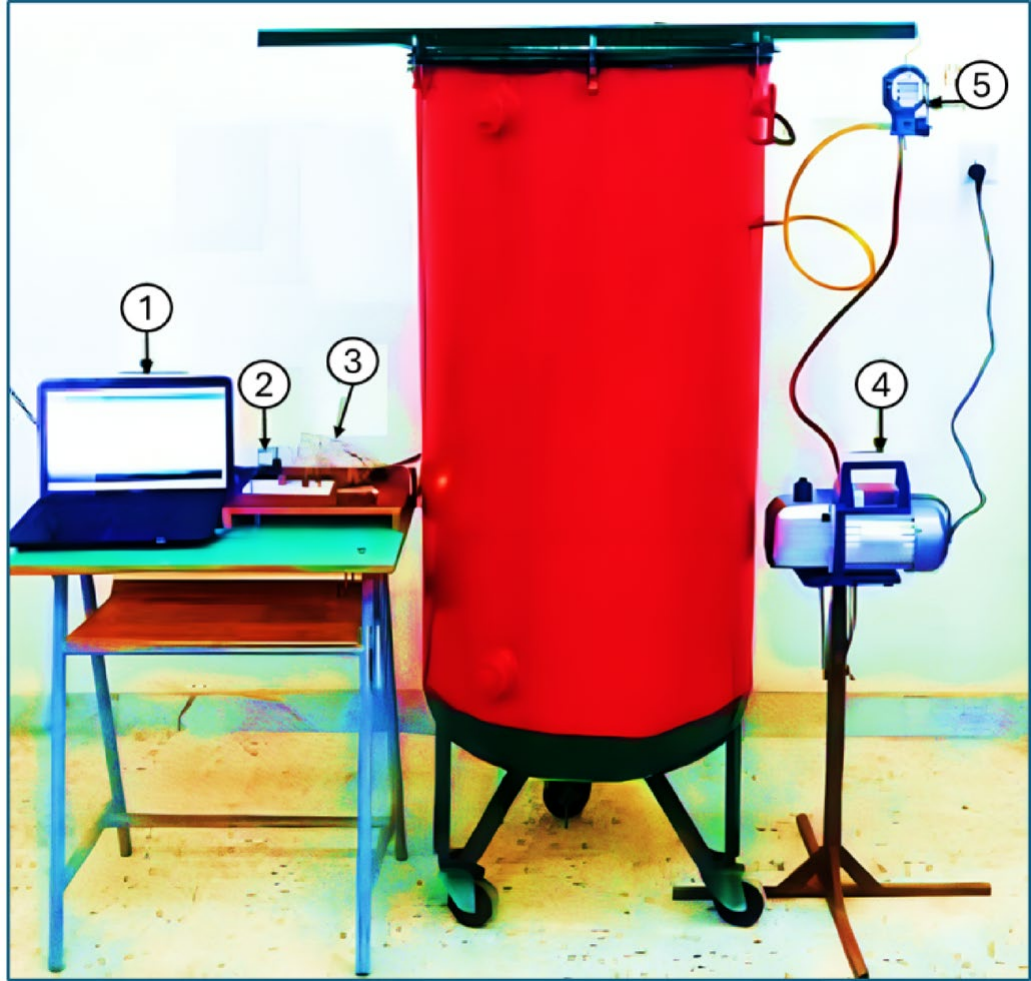
Main components of the DAVD. Whereas (1) Laptop, (2) Arduino uno board, (3) Control unit, (4) Vacuum pump, and (5) Vacuum gauge.

**Fig. 2:**
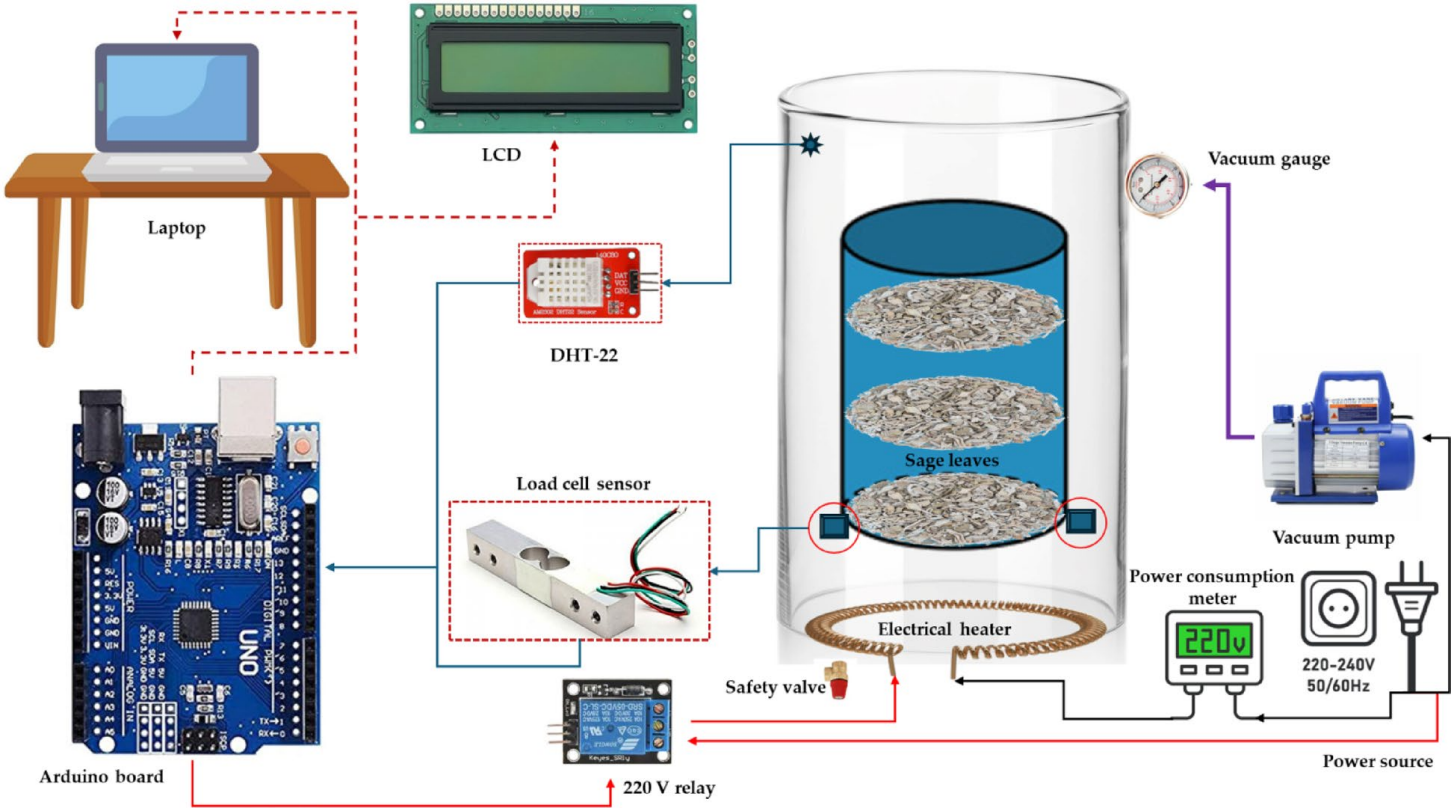
Schematic view showing different components of the DAVD.

As shown in Figs. 1 and 2, the DAVD was manufactured using available materials in local markets in Libya. The DAVD consists of many parts, such as (1) Main body: it was manufactured from reused iron sheets with an average capacity of 200 L; (2) Vacuum pump: A half-hp vacuum pump (model: Vp245, China) regulates the pressure inside the dryer. It can control pressure in a range from − 1 to 3 bars. It is crucial for DAVD as they create and maintain a low-pressure environment, enabling efficient moisture removal at lower temperatures. This preserves heat-sensitive materials, reduces energy consumption, and speeds up drying processes; (3) Vacuum gauge: This instrument measures the pressure inside the dryer and regulates its level. It can measure the pressure in a range from − 0.1 to 6.0 MPa with an accuracy of ± 0.5; (4) Electrical heater: It was used for increasing the air temperature inside the DAVD based on the requirements of the drying process; it has a rated power of 600 watts and is installed in the bottom of the dryer; (5) Load cell sensor: A pair of load cell sensors was used to automatically measure the weight of the sage leaf samples and send the signals to an Arduino board; (6) Relative humidity and temperature sensor: It was used to accurately measure the relative humidity and temperature inside the DAVD; (7) Relay: it was used for controlling the operation of the electrical heater automatically based on the output signals from the DHT-22 and the Arduino Uno board; (8) Control unit: it consists of an Arduino board (model: Arduino Uno, China). It is a microcontroller board based on the ATmega328P. It has 14 digital input/output pins (of which 6 can be used as PWM outputs), 6 analog inputs, a 16 MHz ceramic resonator, a USB connection, a power jack, an ICSP header, and a reset button; (9) Terminal device: The DAVD is integrated with an LCD to present the temperature and humidity signals directly for the user, but during the laboratory experiments, the output signals were transmitted to a laptop and stored in an Excel sheet for subsequent analysis.

### Evaluations processes of the DAVD

#### Drying kinetics

##### Moisture content ($\:{\mu\:}_{d}$)

The initial moisture content of sage leaves in a laboratory is estimated by following the approach defined by AOAC^50^, utilizing the oven-drying method. And it was calculated according to Eq. 1^51,52^.

1 $$\:{\mu\:}_{d}=\left[\frac{{W}_{w}-\:{W}_{d}}{{W}_{d}}\right]\times\:100$$

where, $\:{W}_{w}$ and $\:{W}_{d}\:$is the wet and dry weight of henna leaf sample, respectively in g.

##### Accumulated weight loss

To assess weight loss during the desiccation of sage leaves, the subsequent procedures are implemented: The fresh sage leaves (W_t_) are measured using a precision electronic balance with an accuracy of ± 0.1 g. The fresh sage leaves are thereafter distributed uniformly on the drying trays and positioned in the DAVD. Every 15 min, the weight of the sage leaves sample was recorded using the load cell sensor, and the observed weight (W_t+1_) was documented in an Excel spreadsheet on the laptop. Drying persists until the weight stabilizes (constant weight, W₃). Weight loss is determined using Eq. 2^53,54^.

2 $$\:Weight\:loss\:\left(g\right)={W}_{t}-\:{W}_{t+1}$$

##### Drying rate

The evaluation of drying rates is essential in sectors such as food, pharmaceuticals, and agriculture, as it assesses the time and energy needed to eliminate moisture from substances. It entails the examination of variables such as temperature, humidity, airflow, and material characteristics. Precise estimation guarantees effective drying, maintains product quality, and maximizes energy efficiency in industrial processes. The drying rate was determined using Eq. 3^55^.

3 $$\:Drying\:rate\:({g}_{water}/{g}_{dry\:matter}.h)=\:\frac{Weight\:loss\:\left(g\right)}{\varDelta\:t\:\left(h\right)}$$

##### Moisture ratio (MR)

The moisture ratio calculation is a critical metric in drying processes, indicating the proportion of residual moisture content ($\:{M}_{t}$) in a material relative to its original moisture content ($\:{M}_{0}$). Monitoring drying efficiency, estimating drying durations, and guaranteeing product quality are crucial. Through the analysis of moisture ratio, sectors such as food, agriculture, and pharmaceuticals can enhance drying conditions, minimize energy usage, and avert both over-drying and under-drying. Precise computation aids in sustaining uniformity, extending shelf life, and augmenting total process efficiency^23,24,52,56,57^. The moisture ratio of the dried sage leaf samples was determined using Eq. 4^58^.

4 $$\:MR=\frac{{M}_{t}-{M}_{e}}{{M}_{0}-{M}_{e}}$$

The moisture ratio was utilized to analyze the drying kinetics of sage leaves by appropriate mathematical models. The value of equilibrium moisture content ($\:{M}_{e})$ can be disregarded, as it is rather little relative to the values of $\:{M}_{t}$ and $\:{M}_{0}$. Therefore, the moisture ratio can be represented as shown in Eq. 5^59^.

5 $$\:MR=\frac{{M}_{t}}{{M}_{0}}$$

#### Drying constant (*k*)

The drying constant (*k*) in thin-layer drying is derived from a mixture of many drying transport variables, including mass coefficients, density, specific heat, thermal conductivity, and interfacial heat transfer. The drying constant is determined through the exponential relationship between LnMR and drying time. Furthermore, the drying constant is derived from the same relationship for the DAVD across three operating pressures and three drying temperatures of the sage leaves. While drying constants are crucial for comprehensively defining the drying kinetics of dried product^60,61^, it is vital to consider the diverse transport parameters involved. The drying constant was determined utilizing Eq. 6.

6 $$\:MR=\text{A\:exp}(-k\times\:t)$$

#### Effective moisture diffusivity (EMD)

Fick’s second law of diffusion is an effective framework for comprehending and forecasting moisture diffusion dynamics in materials. Utilizing this idea, researchers and engineers can acquire significant insights into EMD, facilitating progress in several domains dependent on moisture transport regulation^62^. The general equation for mass transfer in a slab shape is shown in Eq. 7^63,64^:

7 $$\:\frac{\partial\:M}{\partial\:t}=\:{D}_{eff}\left(\frac{{\partial\:}^{2}M}{\partial\:{r}^{2}}+\frac{2}{r}\frac{\partial\:M}{\partial\:r}\right)$$

8 $$M\left. {\left( {r,t} \right)} \right|_{{t = 0}} = ~M_{0}$$

9 $$\:{\left.\frac{\partial\:M\left(r,t\right)}{\partial\:r}\right|}_{t=0}=\:0$$

10 $$\:{M\left.\left(R,t\right)\right|}_{t>0}=\:{M}_{e}$$

With the appropriate initial and boundary conditions:

where: *M* is the moisture content, % (w.b.) and *t* is the drying time, s.

The initial boundary condition specifies that moisture is uniformly distributed across the sage leaf sample. The second indicates that the mass transfer is symmetrical relative to the center of the sage leaf sample. The third criterion stipulates that the surface moisture content of the sage leaf sample rapidly attains equilibrium with the ambient air conditions. The values of equilibrium moisture content (*Me*) are rather minimal. Consequently, the third condition can be condensed into Eq. 11.

11 $$\:{\text{M}\left.\left(\text{R},\text{t}\right)\right|}_{\text{t}\:>\:0}=\:0\:\:\:\:\:\:\:\:\:\:\:\:$$

Following the numerical procedure, assume a solution of the following form to separate the variables:

12 $$\:\text{M}\left(r,t\right)=\:\text{F}\left(r\right)\text{*}\text{G}\left(t\right)\:\:\:$$

where F is function of r only, and G is function of t only.

13 $$\:\frac{{M}_{0}-M}{{M}_{0}}=1-\frac{8}{{\pi\:}^{2}}\sum\:_{n=0}^{\infty\:}\left(\frac{1}{{n}^{2}}exp\left(\frac{{-{\uppi\:}}^{2}\times\:{\text{D}}_{\text{e}\text{f}\text{f}}\times\:\text{t}}{4{\text{L}}^{2}}\right)\right)$$

Equation 14 was generated by simplify Eq. 13:

14 $$\:\frac{M}{{M}_{0}}=\frac{8}{{\pi\:}^{2}}\sum\:_{n=0}^{\infty\:}\left(\frac{1}{{n}^{2}}exp\left(\frac{{-{\uppi\:}}^{2}\times\:{\text{D}}_{\text{e}\text{f}\text{f}}\times\:\text{t}}{4{\text{L}}^{2}}\right)\right)$$

Based on the previous moisture ratio (MR) equation and the third boundary condition, Eq. 15 was simplified as follows:

15 $$MR = ~\frac{8}{{\pi ^{2} }} \times \mathop \sum \limits_{{n = 1}}^{\infty } \frac{1}{{n^{2} }}\exp \left( {\frac{{ - \pi ^{2} \times D_{{eff}} \times t}}{{4L^{2} }}} \right)$$

The diffusion coefficients are typically determined by plotting experimental drying data in terms of ln (MR) versus drying time (t), because the plot gives a straight line with a slope as $\:\left[{\pi\:}^{2}\frac{{D}_{eff}}{{R}^{2}}\right]$^65–69^:

16 $$\:\text{M}\text{R}=\:\frac{8}{{{\uppi\:}}^{2}}\times\:\text{A}\:\text{e}\text{x}\text{p}\left(\frac{-{{\uppi\:}}^{2}\times\:{\text{D}}_{\text{e}\text{f}\text{f}}\times\:\text{t}}{4{\text{L}}^{2}}\right)$$

Also, Eq. 17 has been obtained mathematically from Eq. 16,

17 $$\:\text{ln}\left(\text{M}\text{R}\right)=\text{ln}\left(\frac{8}{{{\uppi\:}}^{2}}\right)-\:\left(\frac{{{\uppi\:}}^{2}\times\:{\text{D}}_{\text{e}\text{f}\text{f}}\times\:\text{t}}{4{\text{L}}^{2}}\right)$$

#### Activation energy

The activation energy (E_a_) for the process was determined by applying the Arrhenius equation, following an analogous methodology to that used for calculating the effective diffusion coefficient (D_eff_). This approach establishes the temperature dependence of both parameters, where the natural logarithm of each was plotted against the reciprocal of absolute temperature (1/T). The resulting linear relationships enabled the calculation of E_a_ from the slope of ln(k) versus 1/T, while D_eff_ was similarly derived from its temperature-dependent behavior, demonstrating consistent thermodynamic treatment of both kinetic parameters^70^.

18 $$\:{\text{D}}_{\text{e}\text{f}\text{f}}=\:{\text{D}}_{0}\text{exp}\left(-\frac{{\text{E}}_{\text{a}}}{\text{R}\text{T}}\right)$$

where, $\:{D}_{0}$ is the pre-exponential factor (frequency factor, s⁻¹), R is the universal gas constant (8.314 J/mol.K), and T is the absolute temperature in K.

#### Mathematical modeling of drying process

The experimental data obtained from drying sage leaves at different drying temperatures and operating pressures were evaluated using nine mathematical models (Table 1). Subsequently, non-linear regression analysis was performed using the developed model by Öksüz and Buzrul^71^, to estimate the coefficients of the specified models and statistical metrics (root mean squared error (RMSE), coefficient of determination (*R*^2^ and adjusted coefficient of determination ($\:{R}_{adj.}^{2}$)) presented in Eqs. 19–21. The ideal and most fit mathematical model was determined by the criteria of least RMSE and maximal *R*^2^ and $\:{R}_{adj.}^{2}$^72–74^.

**Table 1:** List of mathematical models used during the current study.

No.	Model name	Model equation*	Reference
1	Aghbashlo	$\:MR=\text{exp}\left(-\frac{{k}_{1}t}{1+{k}_{2}t}\right)$	^71,75^
2	Logarithmic (Asymptotic)	$\:\text{M}\text{R}=\text{a}\text{*}\text{e}\text{x}\text{p}\left(-\text{k}\text{t}\right)+c$	^76–78^
3	Midilli	$\:\text{M}\text{R}=\text{a}\text{*}\text{e}\text{x}\text{p}\left(-\text{k}{\text{t}}^{n}\right)+bt$	
4	Modified midilli I	$\:\text{M}\text{R}=\text{e}\text{x}\text{p}\left(-\text{k}{\text{t}}^{n}\right)+bt$	^79,80^
5	Modified midilli II	$\:\text{M}\text{R}=\text{a}\text{*}\text{e}\text{x}\text{p}\left(-\text{k}{\text{t}}^{n}\right)+b$	^79^
6	Page	$\:\text{M}\text{R}=\text{e}\text{x}\text{p}\left(-\text{k}{\text{t}}^{\text{n}}\right)$	^76–78^
7	Wang-Sigh	$\:MR=1+bt+a{t}^{2}$	
8	Weibullian	$\:\text{M}\text{R}=\text{e}\text{x}\text{p}\left(-{\left(\frac{t}{\alpha\:}\right)}^{\beta\:}\right)$	^79,80^
9	Weibullian I	$\:\text{M}\text{R}={10}^{-{\left(\frac{t}{\delta\:}\right)}^{n}}$	

* MR is the moisture ratio, dimensionless: t is the drying time, min: k_1_, k_2_ and k are the drying constants, min^− 1^: a, b, c, n, ɤ, β and δ are the models constants, dimensionless: RMSE is the root mean square error: R^2^ is the coefficient of determination R^2^_adj_. is the adjusted coefficient of determination. All statistical analysis was conducted at *p* ≤ 0.05.

19 $$\:{R}^{2}=1-\frac{\sum\:{{(MR}_{experimental}-{MR}_{fitted})}^{2}}{\sum\:{({MR}_{experimental}-{MR}_{mean})}^{2}}$$

20 $$\:{R}_{adj.}^{2}=1-\left(1-{R}^{2}\right)*\frac{n-1}{n-p}$$

where $\:{MR}_{experimental}$ and $\:{MR}_{fitted}$ are the observed (experimental) value and fitted value by the model, respectively and $\:{MR}_{mean}$ is the average value of the experimental data in Eq. 19, *n* is the number of data points and *p* is the number of parameters in the model^71^. RMSE value is calculated as follows^71^:

21 $$\:RMSE=\sqrt{\frac{\sum\:{{(MR}_{experimental}-{MR}_{fitted})}^{2}}{n-p}}$$

Note that the term in the numerator of Eq. 19 or Eq. 21 is the sum of squared error (SSE) and the term in the denominator of Eq. 19 is sum of squared total (SST). Regretfully, the denominator of RMSE calculation in certain thin-layer modeling literature only includes *n*; however, it should be *n*—*p* because degrees of freedom is the difference between the experimental data and the model, that is, error.

#### Thermodynamic parameters

Thermodynamic parameters, activation enthalpy (ΔH^‡^) in J/K.mol and activation entropy (ΔS^‡^) in J/K.mol, were estimated using Eq. 22^81,82^.

22 $$\ln \left( {\frac{k}{T}} \right) = \left[ {\left( {\frac{{\ln k_{B} }}{h}} \right) + \left( {\frac{{\Delta S^{\ddag } }}{R}} \right) - \left( {\left( {\frac{{\Delta H^{\ddag } }}{R}} \right) \times \left( {\frac{1}{T}} \right)} \right)} \right]$$

where k_B_ is Boltzmann’s constant (1.38065 × 10^–23^ J/K), h the Planck constant (6.62608 × 10^–34^ J.s), and the notation ^‡^ refers to the state of activated complex.

According to Eq. 22, the regression of ln k/T versus 1/T (K^-1^), gives a straight line with the slope $\left( {\frac{{\Delta H^{\ddag } }}{R}} \right)$ and the interception $\left[ {\left( {\frac{{\ln k_{B} }}{h}} \right) + \left( {\frac{{\Delta S^{\ddag } }}{R}} \right)} \right]$, which allows the determination of ΔH^‡^ and ΔS^‡^, respectively. While the Gibbs free energy of activation (ΔG^‡^) was determined for each temperature using Eq. 23^82^.

23 $$\Delta G^{\ddag } = \Delta H^{\ddag } - T\Delta S^{\ddag }$$

#### Economic analysis

An economic examination of the drying process and dryers is essential for increasing efficiency, minimizing costs, and improving sustainability. Drying is energy-intensive and frequently constitutes a substantial amount of operational costs in sectors such as agriculture, food processing, and manufacturing. By assessing variables like as energy usage, drying duration, equipment expenses, and maintenance, enterprises may pinpoint economical alternatives and enhance profitability. Furthermore, economic analysis aids in the selection of appropriate dryer technology, optimizing capital expenditure against operational savings. It additionally advances environmental objectives by endorsing energy-efficient methods, decreasing waste, and mitigating carbon footprints. This research ultimately secures a competitive advantage by aligning economic and operational performance with market demands^83–86^. The economic analysis of the DAVD was assessed utilizing Eqs. 24–35. Table 2 presents the calculation assumptions for the economic analysis of the DAVD based on economic aspects in Libya.

**Table 2:** Calculation assumptions of economic analysis of the DAVD.

Parameter	Nomenclature	Unit	Value
Interest rate	$\:d$	%	3%
Maintenance cost	$\:{C}_{m}$	USD/year	3% of the annual capital cost
Salvage value	$\:{V}_{a}$	%	8% of the annual capital cost
Operating life	*τ*	year	20 years
Inflation rate	$\:i$	%	2.5%

The annual investment cost of the DAVD is calculated by summing the capital cost (amortized over its lifespan), annual maintenance expenses, energy consumption costs, and other operational expenditures. This calculation helps determine the annual investment cost, aiding in budgeting, cost optimization, and decision-making for efficient and economical dryer selection and operation. The annual investment cost ($\:{C}_{a}$ in USD/year) of the DAVD was calculated using Eq. 24.

24 $$\:{C}_{a}={C}_{ac}+{C}_{m}-{V}_{a}$$

The annual capital cost of the DAVD is calculated by dividing the initial purchase cost by its expected lifespan in years, often incorporating interest or financing rates. Where the annual capital cost ($\:{C}_{ac}$) of the DAVD was calculated according to Eqs. 25 and 26, as follows,

25 $$\:{C}_{ac}={C}_{cc}\times\:{F}_{c}$$

26 $$\:{F}_{c}=\:\frac{{d(1+d)}^{\tau\:}}{{(1+d)}^{\tau\:}-1}$$

where $\:{C}_{cc}$ is the capital cost of the DAVD, $\:{F}_{c}$ is the recovery factor.

The drying cost per kg of sage leaves using the DAVD ($\:{C}_{s}$), was calculated using Eq. 27^87,88^.

27 $$\:{C}_{s}=\frac{{C}_{a}}{{M}_{y}\:}$$

The amount of dried sage leaves using the DAVD per year ($\:{M}_{y}$) is calculated using Eq. 28, where the total amount of sage leaves using the DAVD per batch ($\:{M}_{d}$) was 2 kg. Additionally, the number of days available for drying per year ($\:{D}_{d}$) assumed to be 350 days.

28 $$\:{M}_{y}=\frac{{M}_{d}\times\:D}{{D}_{d}\:}$$

Based on local markets in Libya, the cost of fresh sage leaves ($\:{C}_{fd}$) was about 2.0 USD per kg. And the cost of one kilogram of the dried sage leaves ($\:{C}_{ds}$) was calculated using Eq. 29^87,88^.

29 $$\:{C}_{ds}={C}_{dp}+{C}_{s}$$

where $\:{C}_{dp}$ is the cost of fresh sage leaves per kg of dried product, which is calculated using Eq. 30,

30 $$\:{C}_{dp}={C}_{fd}\times\:\frac{{M}_{f}}{{M}_{d}}$$

where, $\:{M}_{f}$ is the quantity of fresh sage leaves loaded inside the DAVD.

Based on local markets in Libya, the selling price of dried sage leaves ($\:{SP}_{c}$) is about 5.0 USD per kg. Also, the savings obtained per kg of dried sage leaves ($\:{S}_{kg}$) are given by Eq. 31,

31 $$\:{S}_{kg}={SP}_{c}-{C}_{ds}$$

The savings obtained from the DAVD per batch ($\:{S}_{b}$) are given by Eq. 32,

32 $$\:{S}_{b}={S}_{kg}\times\:{M}_{d}$$

While the savings obtained from the DAVD per day ($\:{S}_{d}$) are given by Eq. 33,

33 $$\:{S}_{d}=\frac{{S}_{b}}{D}$$

The savings obtained from the DAVD after “j” number of years is given by Eq. 34,

34 $$\:{S}_{j}={S}_{d}\times\:D\times\:{\left(1+j\right)}^{j-1}$$

The payback time (Ŧ) for the DAVD is calculated using Eq. 35^87,88^.

35 $$T = \frac{{\ln \left[ {1 - \frac{{C_{{cc}} }}{{S_{1} }}\left( {d - i} \right)} \right]}}{{\ln \left( {\frac{{1 + i}}{{1 + d}}} \right)}}$$

where, $\:{S}_{1}$ is the savings obtained from the DAVD after the first year.

## Results and discussions

### Accumulated weight loss and moisture content

**Fig. 3:**
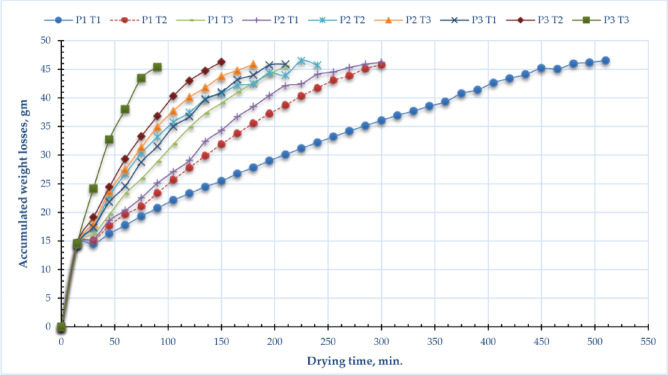
Accumulated weight losses of sage leaves for DAVD at different levels of drying pressures (P1 = atm; P2 = -5 kPa and P3 = -10 kPa) and drying temperatures (T1 = 40, T2 = 50 and T3 = 60 °C)^53,95–97^.

Many crops experience significant weight loss, adversely affecting their quality and reducing profitability. For instance, they may deteriorate in shape or texture, or the color may become compromised^89^. The principal factor contributing to weight loss is the leaching and diffusion process, wherein water-soluble constituents such as vitamins, flavors, minerals, carbs, sugars, and proteins are extricated from plant tissue into the environment^90^. Figure 3 illustrates the cumulative weight losses of sage leaves under varying operating pressures and drying temperatures. The depicted data in the figure indicated that the weight loss of sage leaves remained consistent across various treatments. However, weight loss escalated with rising drying temperatures and operating pressures. The maximum weight loss occurred at an air temperature of 60 °C and an operating pressure of −10 kPa (about 14.6 g), whereas the minimum weight loss was recorded at an air temperature of 40 °C and atmospheric pressure (about 14.4 g). This suggests that weight loss correlates positively with increased power; higher power results in greater weight loss. Kidmose et al.^91^ and Wang et al.^89^ encountered a similar phenomenon. Additionally, Fig. 3 demonstrated that considerable moisture loss occurred during the declining rate period, supporting results from various prior studies^92–94^. Moreover, samples of dried sage leaves attained an equilibrium moisture content ranging from 13.1 to 13.7% (wet basis).

### Moisture ratio

**Fig. 4:**
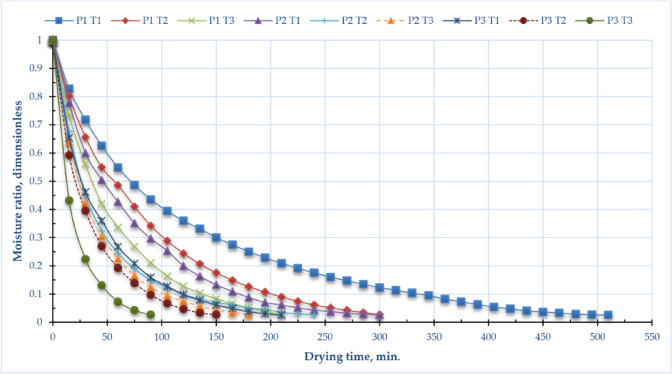
Moisture ratio of sage leaves for DAVD at different levels of drying pressures (P1 = atm; P2 = −5 kPa and P3 = −10 kPa) and drying temperatures (T1 = 40, T2 = 50 and T3 = 60 °C).

Figure 4 illustrates the variation in operating pressure and drying temperatures concerning the moisture ratio of sage leaves. The drying duration necessary to achieve the equilibrium moisture content varied from 90 min to 510 min, contingent upon the operating pressure and drying temperature. Drying sage leaves under atmospheric pressure and 40 °C results in reaching the equilibrium moisture content after 510 min. The drying of sage leaves at an operating pressure of -10 kPa and a temperature of 60 °C resulted in the attainment of equilibrium moisture content after 90 min. The three exhibited a direct correlation between drying temperature and drying time and an inverse correlation between operating pressure and drying time. Reducing the working pressure from atmospheric pressure to -10 kPa decreases the drying time of the final product by about 142.9%, 100%, and 133.3% at drying temperatures of 40 °C, 50 °C, and 60 °C, respectively. This observation aligns with the findings of Beigi^98^ and Kaleta et al.^96^, which demonstrated that elevating the air temperature from 50 to 60 °C during drying enhanced mass transfer, shortened process duration, and decreased energy consumption. Where in vacuum drying, diminished pressure decreases the boiling point of water, facilitating moisture extraction at lower temperatures. This procedure reduces thermal degradation and energy usage. The moisture ratio, indicating the residual moisture content in relation to the initial quantity, diminishes more effectively under vacuum, rendering it a crucial element in enhancing drying kinetics and product quality^24,99^.

Table 3 displays the drying coefficient (k) and determination coefficient (R²) for sage leaves under different operating pressures and drying temperatures. The tabulated data indicated that as the drying air temperature in the DAVD increases, the drying coefficient (k) also rises. Furthermore, the drying coefficient exhibited a significant increase as the system pressure within the dryer was reduced. The value rose from 0.0069 to 0.0164 as the drying temperatures escalated from 40 to 60 °C under atmospheric pressure. Also, the value rose from 0.0124 to 0.0195 as the drying temperatures escalated from 40 to 60 °C at -5 kPa. Additionally, the value rose from 0.0171 to 0.0398 as the drying temperatures escalated from 40 to 60 °C, at − 10 kPa. This aligns with prior research^53,95–97^. No discernible trend can be observed in the coefficient of determination data. At atmospheric pressure, the minimum R^2^ was recorded at 40 °C and the maximum at 50 °C, but at an operating pressure of -10 kPa, the minimum value was noted at 60 °C and the maximum at 50 °C.

**Table 3:** Drying constant (k) and coefficient determination (R^2^) for experimental or observed of sage leaves for DAVD at different levels of drying pressures and drying temperatures.

Coefficient	atm			−5 kPa			−10 kPa		
	40 °C	50 °C	60 °C	40 °C	50 °C	60 °C	40 °C	50 °C	60 °C
k	0.0069	0.0116	0.0164	0.0124	0.0143	0.0195	0.0171	0.0240	0.0398
R^2^	0.9947	0.9987	0.9981	0.9959	0.9734	0.9915	0.9950	0.9964	0.9927

### Drying rate

**Fig. 5:**
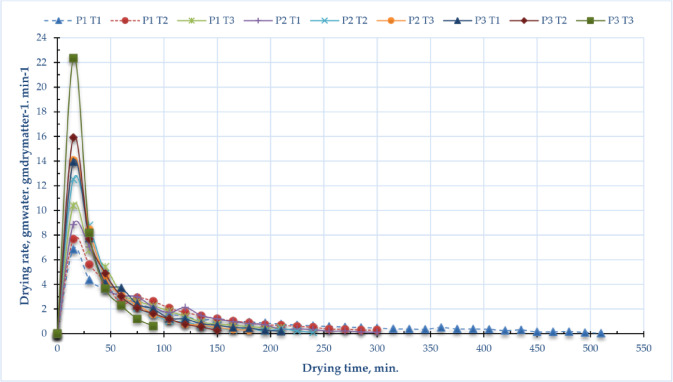
Drying rate of sage leaves for DAVD at different levels of drying pressures (P1 = atm; P2 = −5 kPa and P3 = −10 kPa) and drying temperatures (T1 = 40, T2 = 50 and T3 = 60 °C).

**Fig. 6:**
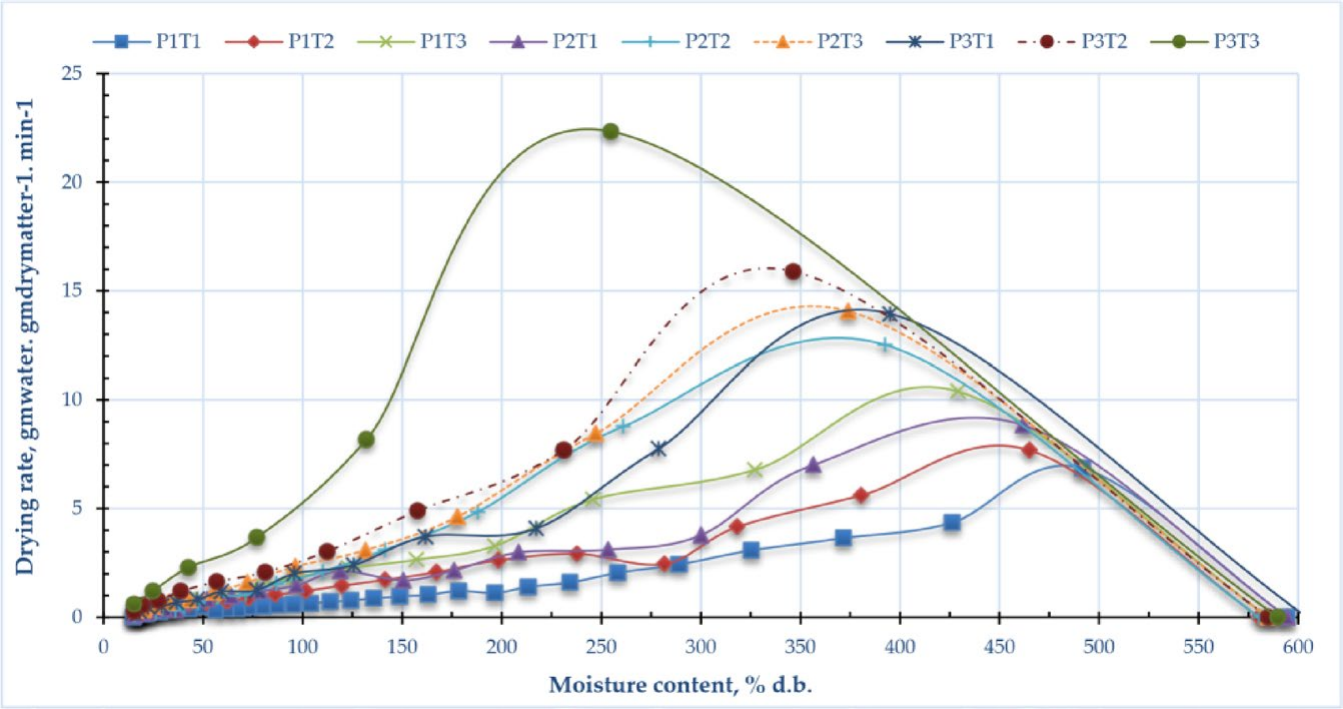
Drying rate with moisture content of sage leaves for DAVD at different levels of drying pressures (P1 = atm; P2 = −5 kPa and P3 = −10 kPa) and drying temperatures (T1 = 40, T2 = 50 and T3 = 60 °C).

Figure 5 illustrates the drying rate of sage leaves at several drying pressures (P1 = atm; P2 = -5 kPa; P3 = -10 kPa) and temperatures (T1 = 40 °C; T2 = 50 °C; T3 = 60 °C). Figure 6 depicts the correlation between drying speeds and moisture content of sage leaves using DAVD at varying drying pressures and temperatures. At the onset of drying (t = 0), the drying rate was zero. During the initial stage, the drying rate reached its maximum due to the rapid evaporation of free (unbound) moisture from the cellular structure. As the process progressed, the depletion of free water led to a gradual decline in the drying rate. This trend continued until the material reached equilibrium moisture content, at which point drying effectively ceased. The elevated drying rates of sage leaf samples dried using DAVD varied from 6.85 to 10.39 kg_water_/kg_dry matter_/h, 8.84 to 14.07 kg_water_/kg_dry matter_/h, and 13.95 to 22.34 kg_water_/kg_dry matter_/h at drying temperatures of 40 to 60 °C, corresponding to operating pressures of atm, -5 kPa, and − 10 kPa, respectively. The data illustrated in Fig. 6 indicates a direct correlation between the drying rate and temperature, as well as between the drying rate and operating pressures, with the maximum drying rate recorded at a temperature of 60 °C and operating pressures of -10 kPa. The drying rate is substantially affected by both drying temperature and operating pressure. Increased drying temperatures expedite the process by supplying additional thermal energy, thereby facilitating moisture evaporation and internal diffusion. The operating pressure substantially influences the drying rate. Decreased pressures lower the boiling point of water, facilitating expedited evaporation at reduced temperatures, thereby enhancing drying efficiency.

**Fig. 7:**
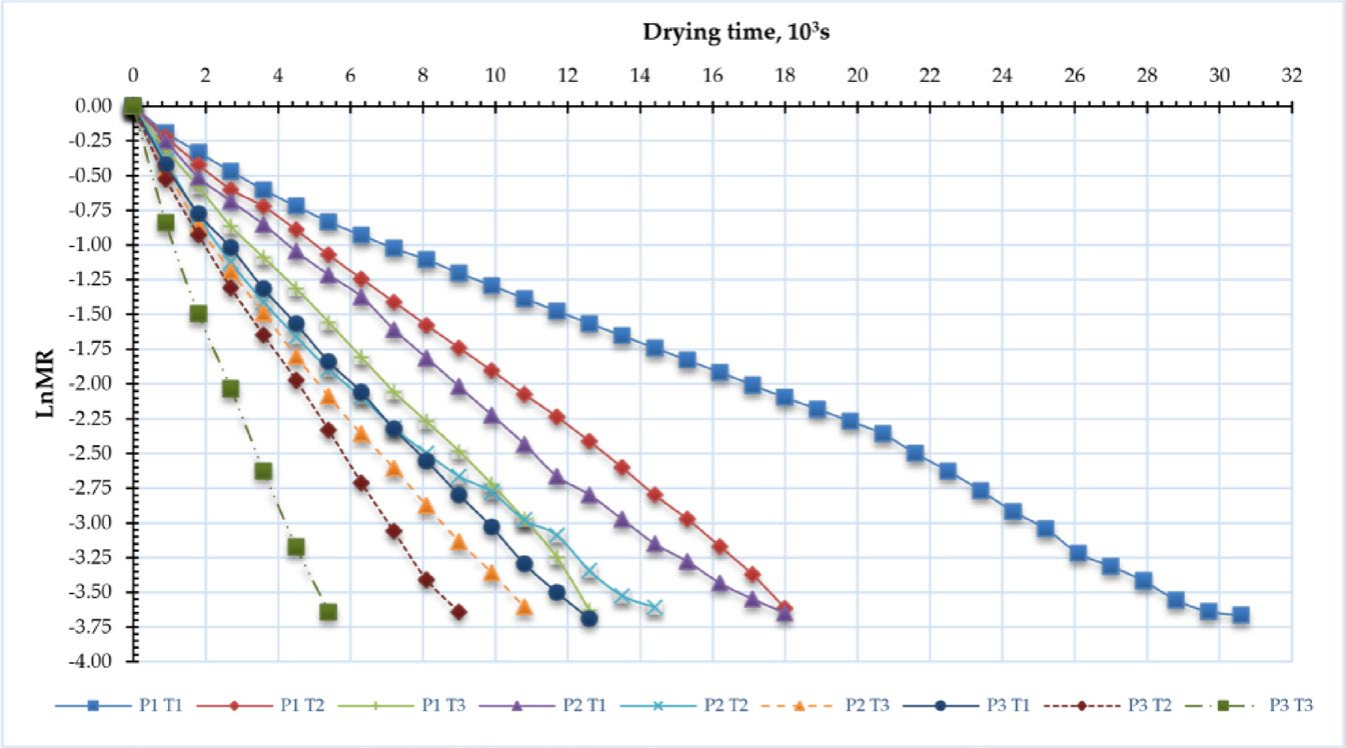
Ln MR with drying time of sage leaves for DAVD at different levels of drying pressures (P1 = atm; P2 = −5 kPa and P3 = −10 kPa) and drying temperatures (T1 = 40, T2 = 50 and T3 = 60 °C).

Figure 7 illustrates the drying rate versus moisture content of sage leaves under different conditions of drying pressures (P1 = atmospheric pressure, P2 = -5 kPa, and P3 = -10 kPa) and temperatures (T1 = 40 °C, T2 = 50 °C, and T3 = 60 °C) in a DAVD (Drying Assisted by Vacuum Drying) system. The graph demonstrates how both reduced pressure and elevated temperature influence the drying kinetics, with lower pressures (P3) and higher temperatures (T3) likely accelerating moisture removal due to enhanced driving forces for evaporation. The trend suggests that the drying rate is highest at the initial stages when free moisture is abundant, then declines as moisture content decreases, eventually stabilizing near equilibrium. This behavior aligns with typical drying curves, where external conditions (pressure and temperature) significantly impact the rate of water removal.

The current study demonstrates that vacuum drying of sage leaves at 60 °C with reduced pressures (-10 kPa) achieves a significantly higher drying rate (22.34 kg_water_/kg_dry matter/h_) compared to conventional drying methods used for other herbs, as shown in Table 4. For instance, heat pump drying of amaranth leaves (5.0), oven drying of rue leaves (3.55), hybrid solar drying of basil (1.7), and even high-temperature convective drying of coriander (2.3) all yielded lower rates. While microwave and oven drying of Vernonia amygdalina leaves reached 20 and 6.0, respectively, the vacuum drying method in this study surpassed these values, highlighting its superior efficiency in moisture removal. This enhanced performance is attributed to the combined effect of elevated temperature and reduced pressure, which accelerates moisture diffusion while preserving product quality, making vacuum drying a promising technique for industrial-scale herb processing.

**Table 4:** Comparison between the achieved drying rate with previous studies.

Ref.	Product	Dryer type and drying conditions	Drying rate (kg_water_/kg_dry matter_/h)
^100^	Amaranth leaves	Heat pump dryer at drying temperature of 56 °C and air speed of 1.41 m/s.	5.0
^101^	Rue leaves	Oven electric dryer at drying temperature of 70 °C	3.55
^24^	Basil leaves	Hybrid solar dryer at drying temperature of 60 °C	1.7
^102^	Vernonia amygdalina leaves	Oven electric dryer at drying temperature of 60 °C	20
^103^	Coriander leaves	Microwave drying at 1000 W	6.0
^103^	Coriander leaves	Convective drying at drying temperature of 120 °C	2.3
Current study	Sage leaves	Vacuum dryer at drying temperature of 60 °C, and operating pressures of atm, −5 kPa, and −10 kPa	22.34

### Effective moisture diffusivity (EMD)

**Fig. 8:**
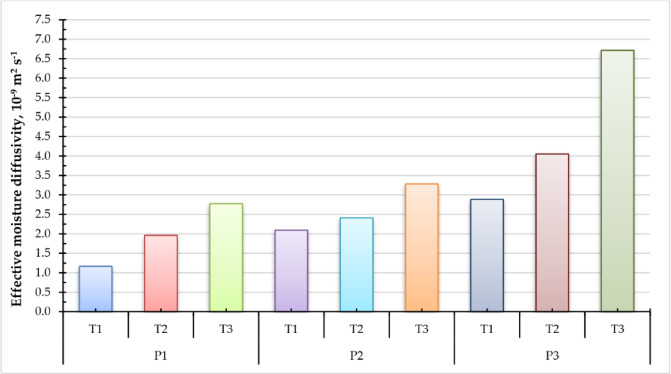
Effective moisture diffusivity of sage leaves for DAVD at different levels of drying pressures (P1 = atm; P2 = −5 kPa and P3 = −10 kPa) and drying temperatures (T1 = 40, T2 = 50 and T3 = 60 °C).

Experimental data of moisture content has converged to construct portraits of ln (MR) over time (t) as depicted in Fig. 7. Where a linear connection between drying time and ln (MR) was discovered. The key experimental parameter typically employed for simulating drying processes is the reduction in sample mass, which is defined as the ratio between the water content at a specific time (t) and the original moisture content^104^. The findings displayed in Fig. 8 demonstrate that the drying time is largely dictated by the internal mass transfer resistance, which is influenced by the existence of a decreasing rate drying phase. Consequently, the EMD values for the drying experiment under various situations are computed using Fick’s second law. The EMD of the different sage samples was measured for different pressure settings and drying temperatures (Fig. 8). When the operating pressure reduced from atm to − 100 kP, the EMD increased from 1.165 × 10^− 9^ to 2.886 × 10^− 9^ m^2^/s, from 1.959 × 10^− 9^ to 4.053 × 10^− 9^ m^2^/s, and from 2.774 × 10^− 9^ to 6.716 × 10^− 9^ m^2^/s, at drying temperatures of 40, 50, and 60 °C, respectively. On the other hand, when the drying temperatures increased from 40 °C to 60 °C, the EMD increased from 1.165 × 10^− 9^ to 2.774 × 10^− 9^ m^2^/s, from 2.094 × 10^− 9^ to 3.283 × 10^− 9^ m^2^/s, and from 2.886 × 10^− 9^ to 6.716 × 10^− 9^ m^2^/s, at operating pressures of atm, – 5 kP, and – 10 kP, respectively. This can be clarified as follows: An increase in air temperature and a reduction in operating pressure will accelerate the movement of water molecules, resulting in a heightened rate of water diffusion. This subsequently enhances the thermal and mass transfer between the solid-liquid film and the heated air. Consequently, the moisture concentration and partial pressure of water vapor on the leaf surface diminish, thereby expediting the internal evaporation, migration, and EMD processes within the sage leaves^105^. Moreover, reducing pressure in a vacuum dryer markedly improves EMD by decreasing the boiling point of water, facilitating moisture evaporation at lower temperatures^106,107^. This enhances the pushing power for moisture extraction and diminishes the barrier to vapor diffusion within the material. The drying process becomes more efficient, especially for heat-sensitive substances, as enhanced diffusivity accelerates moisture transfer and improves overall drying kinetics. The EMD values acquired in the present study exceeded those reported in other research, including Ouahida’s^63^ dried sage leaves, which utilized a convection oven at drying temperatures of 30, 45, and 60 °C, where the EMD varied from 1.1 to 3.7 × 10^–12^ m^2^/s m²/s. Additionally, Ahmed^108^ employed a microwave at three distinct power density levels of 6.7, 10, and 20 W/g for the desiccation of sage leaves, achieving a maximum EMD of 1.73 × 10^− 9^ m^2^/s during the investigation. Doymaz and Karasu^45^ assessed the impact of air temperature on the EMD of sage leaves at temperatures of 45, 50, 55, 60, and 65 °C in a cabinet dryer, concluding that the EMD values were considerably influenced by temperature, ranging from 1.62 × 10⁻⁹ to 5.73 × 10⁻⁹ m²/s.

### Activation energy

**Fig. 9:**
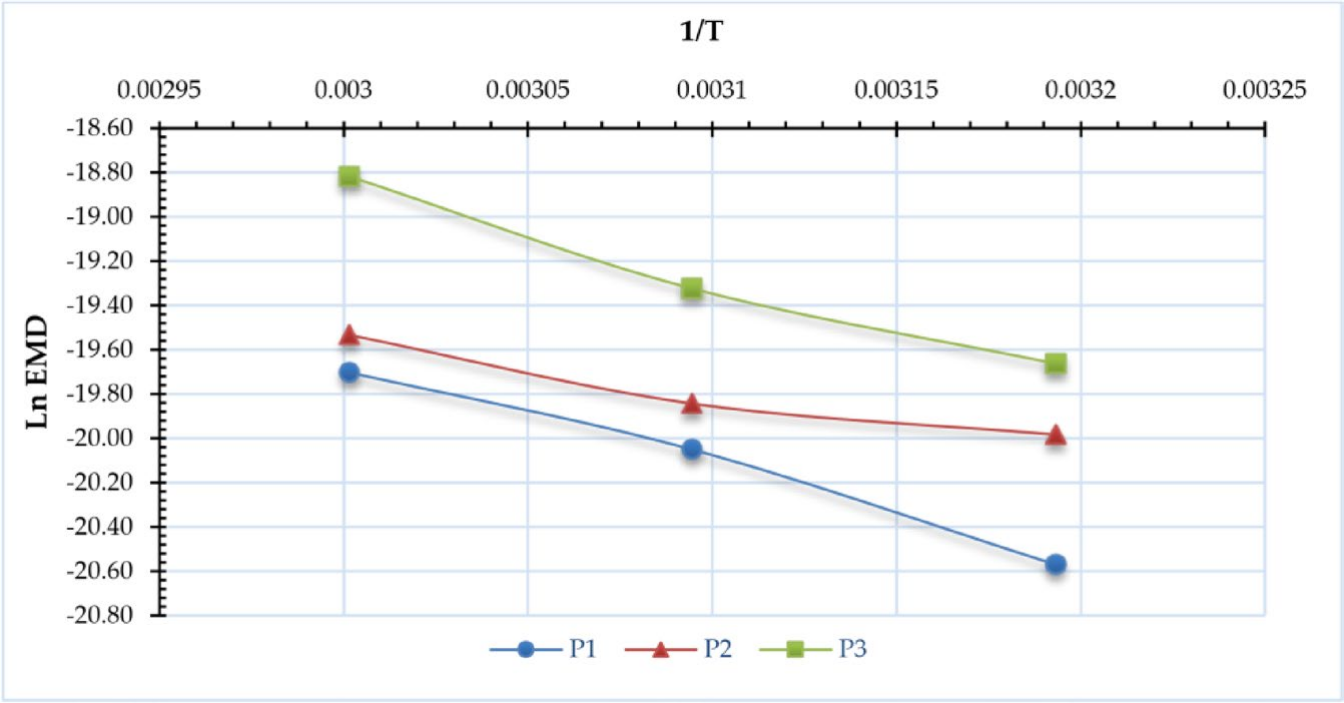
LnEMD vs. 1/T of sage leaves for DAVD at different levels of drying pressures (P1 = atm; P2 = −5 kPa and P3 = −10 kPa).

**Fig. 10:**
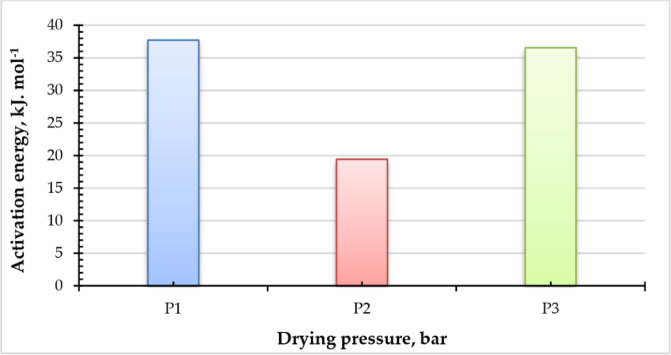
Activation energy vs. drying of sage leaves at different levels of drying pressures (P1 = atm; P2 = −5 kPa and P3 = −10 kPa).

Figure 9 illustrates the correlation between ln (EMD) and (1/T). Also, Fig. 10 illustrates the activation energy of various sage leaf samples at distinct operating pressures, determined to determine the lowest energy required for drying to occur. Across the three tested operating pressures (P1 = atmospheric pressure, P2 = -5 kPa, P3 = -10 kPa), the activation energy exhibited a non-monotonic trend. Specifically, it decreased from 37.7 kJ/mol (P1) to 19.4 kJ/mol (P2) as the pressure was reduced from atmospheric to -5 kPa but then increased to 36.5 kJ/mol (P3) when the pressure was further lowered to -10 kPa. This suggests that the relationship between pressure and activation energy is not linear, possibly indicating competing mechanisms—such as enhanced moisture removal at moderate vacuum (-5 kPa) followed by altered mass transfer dynamics at higher vacuum levels (-10 kPa). The results are inferior to those reported by Ouahida^63^, who dried sage leaves in a convection oven at three temperatures: 30, 45, and 60 °C, with an activation energy of 63.45 kJ/mol. And Doymaz and Karasu^45^ assessed the impact of air temperature on the activation energy of sage leaves at 45, 50, 55, 60, and 65 °C in a cabinet dryer, concluding that the activation energy was 52.52 kJ/mol for these temperatures.

### Mathematical modeling

**Fig. 11:**
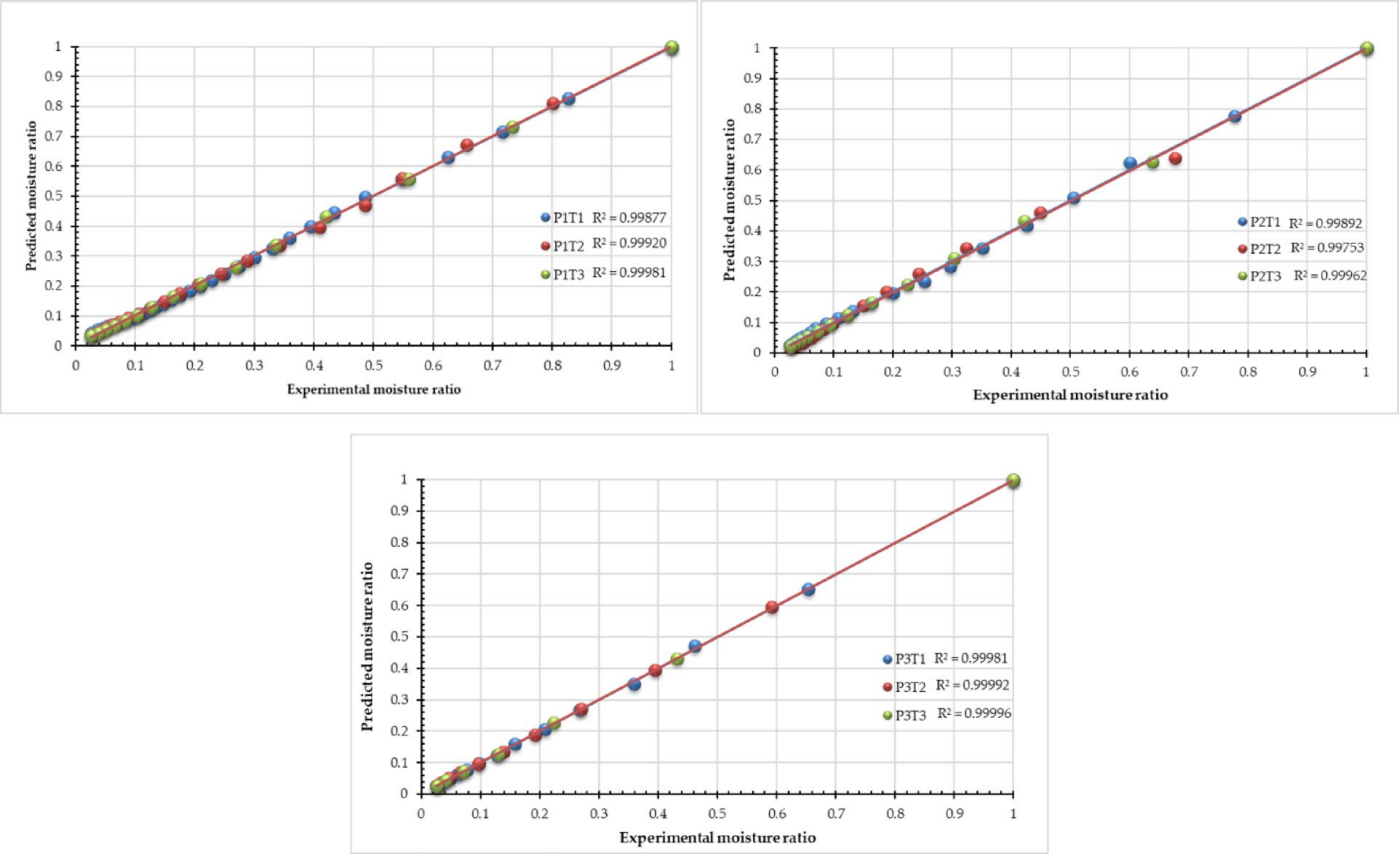
Observed and predicted MR at different drying time for the best model (Page model) of sage leaves at different levels of drying pressures (P1 = atm; P2 = −5 kPa and P3 = −10 kPa) and drying temperatures (T1 = 40, T2 = 50 and T3 = 60 °C).

The moisture content data collected during the drying experiment were transformed into the moisture ratio (MR) and fitted to the nine mathematical models presented in Table 5. Also, Table 5 presents the statistical outcomes of the various mathematical models, encompassing the drying model coefficients and the metrics employed to assess goodness of fit, specifically R^2^, $\:{R}_{adj.}^{2}$ and RSME. Typically, R^2^ and $\:{R}_{adj.}^{2}$ values above 0.99, while RMSE values fell below 0.01. The R^2^ and $\:{R}_{adj.}^{2}$values of the Aghbashlo model were 0.81 and 0.78 at an operating pressure of -5 kPa for drying temperatures of 50 and 60 °C, respectively, and were 0.73 at an operating pressure of -10 kPa for a drying temperature of 40 °C. Figure 11 illustrates the comparison between the experimental moisture ratio at various drying temperatures and the predictions made by the Page model. The dots in Fig. 11 closely align along a 45-degree line, demonstrating a strong correlation between computed and experimental data, indicating that the Page model effectively characterizes the drying behavior of sage leaves. The Page model is deemed appropriate for characterizing the thin layer drying of sage leaves.

**Table 5:** Mathematical models’ constants values and goodness of fit indices results of Sage leaves at different levels of drying pressures and drying temperatures.

MMs	Pressure	T, ºC	Parameters	Models’ constants values				Goodness of fit indices		
				Values	S.E.	p-value	Sign. – Insign.	RMSE	R^2^	R^2^_adj_.
Aghbashlo	atm	40	k_1_	0.01011	0.00028	2.76*10^− 28^	Sign.	0.017960	0.994832	0.994676
			k_2_	0.00134	0.00016	1.84*10^− 9^	Sign.			
		50	k_1_	0.01297	0.00029	1.02*10^− 20^	Sign.	0.011982	0.998200	0.998105
			k_2_	0.00065	0.00018	0.00168	Sign.			
		60	k_1_	0.01994	0.00033	2.77*10^− 17^	Sign.	0.007374	0.999389	0.999342
			k_2_	0.00135	0.00020	1.34*10^− 5^	Sign.			
	−5kPa	40	k_1_	0.01552	0.00040	1.54*10^− 19^	Sign.	0.013379	0.997707	0.997587
			k_2_	0.00102	0.00024	0.00034	Sign.			
		50	k_1_	0.01062	0.00294	0.00254	Sign.	0.118694	0.815249	0.802932
			k_2_	−0.00417	0.00210	0.06548	**Insign.**			
		60	k_1_	0.01029	0.00366	0.01688	Sign.	0.141263	0.780865	0.760944
			k_2_	−0.00556	0.00273	0.06636	**Insign.**			
	−10kPa	40	k_1_	0.02732	0.00077	2.43*10^− 14^	Sign.	0.011646	0.998368	0.998242
			k_2_	0.00339	0.00043	2.86*10^− 6^	Sign.			
		50	k_1_	0.01024	0.00452	0.04958	Sign.	0.165145	0.730648	0.700720
			k_2_	−0.00667	0.00346	0.08580	**Insign.**			
		60	k_1_	0.06047	0.00148	1.67*10^− 7^	Sign.	0.006348	0.999724	0.999669
			k_2_	0.00650	0.00082	0.00052	Sign.			
Logarithmic (Asymptotic)	atm	40	k	0.00827	0.00031	2.18E-23	Sign.	0.021249	0.992986	0.992548
			a	0.90354	0.01369	8.97*10^− 36^	Sign.			
			c	0.03119	0.00818	0.00059	Sign.			
		50	k	0.01195	0.00032	1.61*10^− 18^	Sign.	0.012031	0.998281	0.998090
			a	0.96336	0.00943	2.45*10^− 26^	Sign.			
			c	0.00878	0.00715	0.23550	**Insign.**			
		60	k	0.01879	0.00050	7.60*10^− 14^	Sign.	0.010929	0.998762	0.998555
			a	0.96278	0.00979	8.14*10^− 19^	Sign.			
			c	0.02185	0.00669	0.00675	Sign.			
	-5kPa	40	k	0.01416	0.00043	1.42*10^− 17^	Sign.	0.014598	0.997414	0.997127
			a	0.95609	0.01157	1.12*10^− 24^	Sign.			
			c	0.01477	0.00707	0.05118	**Insign.**			
		50	k	0.02620	0.00079	1.09*10^− 14^	Sign.	0.014370	0.997473	0.997111
			a	0.94357	0.01316	2.28*10^− 19^	Sign.			
			c	0.04461	0.00547	1.10*10^− 6^	Sign.			
		60	k	0.02838	0.00096	4.82*10^− 11^	Sign.	0.014270	0.997967	0.997560
			a	0.94914	0.01365	9.22*10^− 15^	Sign.			
			c	0.03712	0.00719	0.00042	Sign.			
	-10kPa	40	k	0.02407	0.00108	3.98*10^− 11^	Sign.	0.019617	0.995724	0.995012
			a	0.93697	0.01795	1.61*10^− 15^	Sign.			
			c	0.03576	0.00933	0.00238	Sign.			
		50	k	0.03159	0.00144	1.93*10^− 8^	Sign.	0.017654	0.997264	0.996580
			a	0.95253	0.01765	1.54*10^− 11^	Sign.			
			c	0.03155	0.01025	0.01514	Sign.			
		60	k	0.05576	0.00276	3.53*10^− 5^	Sign.	0.016304	0.998545	0.997818
			a	0.96192	0.01849	8.18*10^− 7^	Sign.			
			c	0.03347	0.01111	0.03946	Sign.			
Midilli	atm	40	k	0.02690	0.00126	4.28*10^− 20^	Sign.	0.005193	0.999594	0.999555
			a	1.00617	0.00497	5.81*10^− 50^	Sign.			
			b	-0.00006	0.00001	1.18*10^− 8^	Sign.			
			n	0.75487	0.00939	1.52*10^− 37^	Sign.			
		50	k	0.02014	0.00127	1.25*10^− 11^	Sign.	0.005718	0.999633	0.999569
			a	0.99810	0.00554	2.44*10^− 29^	Sign.			
			b	-7.74*10^− 5^	1.82*10^− 5^	0.00053	Sign.			
			n	0.88047	0.01409	1.58*10^− 21^	Sign.			
		60	k	0.02817	0.00127	1.71*10^− 10^	Sign.	0.004076	0.999842	0.999799
			a	1.00105	0.00403	5.58*10^− 22^	Sign.			
			b	−3.11*10^− 5^	1.94*10^− 5^	0.13685	**Insign.**			
			n	0.89168	0.01113	1.43*10^− 16^	Sign.			
	-5kPa	40	k	0.02398	0.00221	4.48*10^− 9^	Sign.	0.009033	0.999065	0.998900
			a	0.99816	0.00880	6.39*10^− 26^	Sign.			
			b	−3.77*10^− 5^	2.35*10^− 5^	0.12687	**Insign.**			
			n	0.87618	0.02078	1.21*10^− 18^	Sign.			
		50	k	0.04293	0.00428	1.75*10^− 7^	Sign.	0.010677	0.998704	0.998406
			a	1.00463	0.01060	7.49*10^− 20^	Sign.			
			b	0.00011	2.85*10^− 5^	0.00184	Sign.			
			n	0.85296	0.02492	3.99*10^− 14^	Sign.			
		60	k	0.04600	0.00261	2.79*10^− 8^	Sign.	0.005470	0.999731	0.999642
			a	1.00140	0.00545	2.12*10^− 17^	Sign.			
			b	5.41*10^− 5^	2.67*10^− 5^	0.07355	**Insign.**			
			n	0.85422	0.01509	8.45*10^− 13^	Sign.			
	-10kPa	40	k	0.04876	0.00196	5.00*10^− 11^	Sign.	0.004085	0.999830	0.999784
			a	1.00025	0.00406	6.22*10^− 22^	Sign.			
			b	−1.97*10^− 5^	1.74*10^− 5^	0.28183	**Insign.**			
			n	0.80409	0.01026	1.82*10^− 16^	Sign.			
		50	k	0.05587	0.00144	2.00*10^− 9^	Sign.	0.002339	0.999958	0.999940
			a	0.99998	0.00233	9.95*10^− 17^	Sign.			
			b	−4.62*10^− 5^	1.69*10^− 5^	0.0294	Sign.			
			n	0.82563	0.00724	1.05*10^− 12^	Sign.			
		60	k	0.09224	0.00412	0.00020	Sign.	0.003040	0.999962	0.999924
			a	1.00004	0.00304	6.19*10^− 8^	Sign.			
			b	−7.71*10^− 6^	4.62*10^− 5^	0.87813	**Insign.**			
			n	0.81636	0.01462	1.27*10^− 5^	Sign.			
Modified midilli I	atm	40	k	0.02584	0.00088	1.17*10^− 24^	Sign.	0.005240	0.999574	0.999547
			b	−0.00006	0.00001	1.17*10^− 8^	Sign.			
			n	0.76197	0.00751	1.02*10^− 41^	Sign.			
		50	k	0.02042	0.00096	3.06*10^− 14^	Sign.	0.005576	0.999631	0.999590
			b	−7.89*10^− 5^	1.72*10^− 5^	0.00024	Sign.			
			n	0.87773	0.01130	3.40*10^− 24^	Sign.			
		60	k	0.02799	0.00101	3.14*10^− 12^	Sign.	0.003915	0.999841	0.999815
			b	-3.03*10^− 5^	1.83*10^− 5^	0.12456	**Insign.**			
			n	0.89301	0.00950	1.41*10^− 18^	Sign.			
	-5kPa	40	k	0.02427	0.00170	3.08*10^− 11^	Sign.	0.008790	0.999062	0.998958
			b	-3.87*10^− 5^	2.24*10^− 5^	0.10193	**Insign.**			
			n	0.87383	0.01702	5.66*10^− 21^	Sign.			
		50	k	0.04206	0.00359	1.29*10^− 8^	Sign.	0.010365	0.998685	0.998497
			b	0.00011	2.75*10^− 5^	0.00112	Sign.			
			n	0.85707	0.02235	1.40*10^− 15^	Sign.			
		60	k	0.04571	0.00223	1.66*10^− 9^	Sign.	0.005209	0.999729	0.999675
			b	5.47*10^− 5^	2.53*10^− 5^	0.05581	**Insign.**			
			n	0.85556	0.01348	2.29*10^− 14^	Sign.			
	−10kPa	40	k	0.04871	0.00165	1.43*10^− 12^	Sign.	0.003912	0.999830	0.999802
			b	−1.96*10^− 5^	1.66*10^− 5^	0.26035	**Insign.**			
			n	0.80434	0.00909	2.91*10^− 18^	Sign.			
		50	k	0.05587	0.00124	6.68*10^− 11^	Sign.	0.002188	0.999958	0.999947
			b	−4.62*10^− 5^	1.58*10^− 5^	0.01911	Sign.			
			n	0.82563	0.00646	1.58*10^− 14^	Sign.			
		60	k	0.09223	0.00349	1.22*10^− 5^	Sign.	0.002633	0.999962	0.999943
			b	−7.66*10^− 5^	4.00*10^− 5^	0.85742	**Insign.**			
			n	0.81640	0.01253	3.32*10^− 7^	Sign.			
Modified midilli I I	atm	40	k	0.02671	0.00132	2.03*10^− 19^	Sign.	0.005578	0.999532	0.999487
			a	1.04767	0.00973	1.81*10^− 41^	Sign.			
			b	−0.04120	0.00667	7.38*10^− 7^	Sign.			
			n	0.74589	0.01125	5.74*10^− 35^	Sign.			
		50	k	0.02027	0.00121	5.01*10^− 12^	Sign.	0.005497	0.999661	0.999601
			a	1.03063	0.01035	5.79*10^− 22^	Sign.			
			b	−0.03196	0.00759	0.00059	Sign.			
			n	0.87062	0.01485	4.59*10^− 21^	Sign.			
		60	k	0.02810	0.00129	2.15*10^− 10^	Sign.	0.004148	0.999836	0.999792
			a	1.00863	0.00724	3.26*10^− 19^	Sign.			
			b	−0.00759	0.00530	0.17953	**Insign.**			
			n	0.89017	0.01260	5.70*10^− 16^	Sign.			
	−5kPa	40	k	0.02437	0.00220	3.45*10^− 9^	Sign.	0.008828	0.999107	0.998949
			a	1.01421	0.01358	7.60*10^− 23^	Sign.			
			b	-0.01545	0.00877	0.09633	**Insign.**			
			n	0.86776	0.02238	4.95*10^− 18^	Sign.			
		50	k	0.04050	0.00387	1.07*10^− 7^	Sign.	0.009450	0.998985	0.998751
			a	0.97358	0.01138	2.84*10^− 19^	Sign.			
			b	0.03022	0.00556	0.00011	Sign.			
			n	0.88081	0.02567	3.86*10^− 14^	Sign.			
		60	k	0.04542	0.00256	2.62*10^− 8^	Sign.	0.005223	0.999755	0.999673
			a	0.98892	0.00761	4.83*10^− 16^	Sign.			
			b	0.01231	0.00506	0.03775	Sign.			
			n	0.86246	0.01647	1.69*10^− 12^	Sign.			
	−10kPa	40	k	0.04883	0.00198	5.60*10^− 11^	Sign.	0.004080	0.999830	0.999784
			a	1.00542	0.00647	9.75*10^− 20^	Sign.			
			b	-0.00513	0.00456	0.28461	**Insign.**			
			n	0.80190	0.01164	7.46*10^− 16^	Sign.			
		50	k	0.05599	0.00142	1.78*10^− 9^	Sign.	0.002290	0.999960	0.999942
			a	1.00893	0.00412	5.00*10^− 15^	Sign.			
			b	−0.00890	0.00323	0.02822	Sign.			
			n	0.82169	0.00808	2.35*10^− 12^	Sign.			
		60	k	0.09220	0.00427	0.00022	Sign.	0.003044	0.999962	0.999924
			a	1.00079	0.00624	5.35*10^− 7^	Sign.			
			b	−0.00075	0.00535	0.89773	**Insign.**			
			n	0.81622	0.01724	2.08*10^− 5^	Sign.			
Page	Atm	40	k	0.02139	0.00091	3.69*10^− 22^	Sign.	0.008989	0.998706	0.998666
			n	0.80779	0.00830	3.61*10^− 42^	Sign.			
		50	k	0.01750	0.00088	3.85*10^− 14^	Sign.	0.008210	0.999155	0.999111
			n	0.91907	0.01079	5.20*10^− 26^	Sign.			
		60	k	0.02687	0.00077	2.95*10^− 14^	Sign.	0.004177	0.999804	0.999789
			n	0.90502	0.00663	6.59*10^− 22^	Sign.			
	−5kPa	40	k	0.02241	0.00127	3.29*10^− 13^	Sign.	0.009280	0.998897	0.998839
			n	0.89547	0.01245	1.30*10^− 24^	Sign.			
		50	k	0.05149	0.00466	1.31*10^− 8^	Sign.	0.014270	0.997330	0.997152
			n	0.79730	0.02163	3.93*10^− 16^	Sign.			
		60	k	0.04888	0.00205	8.17*10^− 11^	Sign.	0.005967	0.999609	0.999573
			n	0.83465	0.01043	1.45*10^− 16^	Sign.			
	−10kPa	40	k	0.04743	0.00123	8.46*10^− 15^	Sign.	0.003975	0.999810	0.999795
			n	0.81237	0.00620	1.13*10^− 21^	Sign.			
		50	k	0.05343	0.00121	7.56*10^− 12^	Sign.	0.002996	0.999911	0.999901
			n	0.84013	0.00580	1.80*10^− 16^	Sign.			
		60	k	0.09174	0.00216	1.36*10^− 7^	Sign.	0.002366	0.999962	0.999954
			n	0.81831	0.00688	7.98*10^− 10^	Sign.			
Wang-Sigh	Atm	40	b	−0.00528	0.00019	3.37*10^− 24^	Sign.	0.085043	0.884143	0.880632
			a	7.05*10^− 6^	4.84*10^− 7^	6.24*10^− 16^	Sign.			
		50	b	−0.00840	0.00029	4.64*10^− 17^	Sign.	0.059109	0.956198	0.953893
			a	1.81*10^− 5^	1.24*10^− 6^	8.41*10^− 12^	Sign.			
		60	b	−0.01228	0.00056	1.11*10^− 11^	Sign.	0.066352	0.950551	0.946747
			a	3.83*10^− 5^	3.31*10^− 6^	3.20*10^− 8^	Sign.			
	−5kPa	40	b	−0.00910	0.00036	4.08*10^− 16^	Sign.	0.071964	0.933662	0.930170
			a	2.06*10^− 5^	1.51*10^− 6^	2.81*10^− 11^	Sign.			
		50	b	−0.01255	0.00078	7.37*10^− 11^	Sign.	0.113071	0.832339	0.821161
			a	3.78*10^− 5^	4.08*10^− 6^	1.35*10^− 7^	Sign.			
		60	b	−0.01566	0.00097	5.43*10^− 9^	Sign.	0.092675	0.905685	0.897110
			a	6.02*10^− 5^	6.71*10^− 6^	2.16*10^− 6^	Sign.			
	−10kPa	40	b	-0.01345	0.00080	3.09*10^− 10^	Sign.	0.094699	0.892060	0.883757
			a	4.44*10^− 5^	4.73*10^− 6^	3.65*10^− 7^	Sign.			
		50	b	−0.01827	0.00120	1.02*10^− 7^	Sign.	0.088043	0.923444	0.914937
			a	8.25*10^− 5^	9.89*10^− 6^	1.58*10^− 5^	Sign.			
		60	b	−0.03057	0.00275	0.00010	Sign.	0.096813	0.935890	0.923068
			a	0.00023	3.66*10^− 5^	0.00156	Sign.			
Weibullian	Atm	40	β	0.80779	0.00830	3.61*10^− 42^	Sign.	0.008989	0.998706	0.998666
			α	116.70	0.91973	5.87*10^− 46^	Sign.			
		50	β	0.91907	0.01079	5.20*10^− 26^	Sign.	0.008210	0.999155	0.999111
			α	81.61	0.64681	3.03*10^− 29^	Sign.			
		60	β	0.90502	0.00663	6.59*10^− 22^	Sign.	0.004177	0.999804	0.999789
			α	54.40	0.27263	4.74*10^− 24^	Sign.			
	−5kPa	40	β	0.89547	0.01245	1.30*10^− 24^	Sign.	0.009280	0.998897	0.998839
			α	69.53	0.68687	1.98*10^− 27^	Sign.			
		50	β	0.79730	0.02163	3.93*10^− 16^	Sign.	0.014270	0.997330	0.997152
			α	41.29	0.89771	1.46*10^− 17^	Sign.			
		60	β	0.83465	0.01043	1.45*10^− 16^	Sign.	0.005967	0.999609	0.999573
			α	37.20	0.34647	5.72*10^− 18^	Sign.			
	−10kPa	40	β	0.81237	0.00620	1.13*10^− 21^	Sign.	0.003975	0.999810	0.999795
			α	42.64	0.25012	3.67*10^− 23^	Sign.			
		50	β	0.84013	0.00580	1.80*10^− 16^	Sign.	0.002996	0.999911	0.999901
			α	32.68	0.16395	1.02*10^− 17^	Sign.			
		60	β	0.81831	0.00688	7.98*10^− 10^	Sign.	0.002366	0.999962	0.999954
			α	18.525	0.11210	1.54*10^− 10^	Sign.			
Weibullian I	atm	40	n	0.80779	0.00830	3.61*10^− 42^	Sign.	0.008989	0.998706	0.998666
			δ	327.68	3.32442	2.39*10^− 42^	Sign.			
		50	n	0.91907	0.01079	5.20*10^− 26^	Sign.	0.008210	0.999155	0.999111
			δ	202.24	2.1816	1.05*10^− 26^	Sign.			
		60	n	0.90501	0.00663	6.59*10^− 22^	Sign.	0.004177	0.999804	0.999789
			δ	136.73	0.91560	2.05*10^− 22^	Sign.			
	−5kPa	40	n	0.89547	0.01245	1.30*10^− 24^	Sign.	0.009280	0.998897	0.998839
			δ	176.48	2.26408	2.81*10^− 25^	Sign.			
		50	n	0.79730	0.02163	3.93*10^− 16^	Sign.	0.014270	0.997330	0.997152
			δ	117.52	3.01000	1.68*10^− 16^	Sign.			
		60	n	0.83465	0.01043	1.45*10^− 16^	Sign.	0.005967	0.999609	0.999573
			δ	101.05	1.16095	5.74*10^− 17^	Sign.			
	−10kPa	40	n	0.81237	0.00620	1.13*10^− 21^	Sign.	0.003975	0.999810	0.999795
			δ	119.03	0.85638	5.21*10^− 22^	Sign.			
		50	n	0.84013	0.00580	1.80*10^− 16^	Sign.	0.002996	0.999911	0.999901
			δ	88.19	0.54987	7.24*10^− 17^	Sign.			
		60	n	0.81831	0.00688	7.98*10^− 10^	Sign.	0.002366	0.999962	0.999954
			δ	51.33	0.34393	2.56*10^− 10^	Sign.			

*MMs* mathematical models, *T* drying temperature, *ºC: k*_1_, k_2_ *and k* drying constants, *min*^− 1^; a, b, c, n, α, β and δ: mathematical models constants or parameters, dimensionless, *S.E* Standard error, *Sign. – Insign.* Significant – Insignificant at *p* ≤ 0.05, *RMSE*E The root mean square error, *R*^2^ The coefficient of determination, *R*^2^_adj_. The adjusted coefficient of determination.

### Thermodynamic properties of sage leaves

Equations 4 and 5 are applied to determine these parameters which are shown in Table 6. The values of ΔG^‡^ corresponds to the average for the different drying temperatures. The results for all assays (ΔH^‡^ > 0; ΔS^‡^ < 0 and ΔG^‡^ > 0) indicate that the drying process of sage leaves is endothermic and not a spontaneous process^109,110^. Activation enthalpy (ΔH‡) in the context of drying refers to the minimum energy required to initiate the process of moisture removal. This energy is needed to break the intermolecular bonds between water molecules and the material being dried, allowing water to transition from a liquid to a vapor phase. Higher activation enthalpy means more energy is needed to start the drying process, which can affect the efficiency and rate of drying^111,112^. Activation entropy (ΔS‡) describes the change in the degree of molecular disorder or randomness as the system transitions from the initial state (wet material) to the transition state (where water molecules are being released)^113,114^Gibbs free energy of activation (ΔG‡) combines the effects of both activation enthalpy and activation entropy (ΔG‡ = ΔH‡ - TΔS‡) to provide a comprehensive measure of the energy barrier that must be overcome for drying to occur. It determines the overall spontaneity and rate of the drying process at a given temperature. A lower Gibbs free energy of activation means the drying process is more favorable and can proceed more readily, while a higher value indicates a greater energy barrier, making drying less efficient. Understanding these parameters helps in optimizing drying conditions for different materials^112,115,116^. Activation enthalpy (ΔH‡) values (Table 6) decrease with increasing drying temperature and decreasing the operating pressure, as higher temperatures markedly increase the excitation of the product’s water molecules compared to lower temperatures, hence diminishing the order of the water-product system^114,117,118^. The activation entropy (ΔS‡), a thermodynamic parameter reflecting the molecular disorder during water-product interactions^113,114^, exhibited temperature-independent behavior across the tested drying conditions. Contrary to typical expectations where entropy increases with temperature, ΔS‡ maintained consistent values despite variations in drying temperature. This remarkable invariance suggests the existence of a compensating mechanism in the system’s free energy balance, where enthalpy and entropy changes offset each other to maintain thermodynamic stability throughout the drying process^113,114^Negative entropy values are attributed to chemical adsorption and/or structural modifications of the adsorbent^118,119^. Gibbs free energy of activation (ΔG‡) (Table 6) indicates an endergonic reaction, requiring the infusion of energy into the air for the drying process of the product to occur. Comparable trends have been observed in investigations^112,115,116,118^.

**Table 6:** Thermodynamic properties of Sage leaves for developing automatic vacuum drying system at drying pressures and drying temperatures.

Temperature	Pressure	ΔH^‡^, kJ mol^− 1^	ΔS^‡^, KJ mol^− 1^ K^− 1^	ΔG^‡^, kJ mol^− 1^
40 °C	atm	35.10	−0.162	85.72
	−5 kPa	16.82	−0.108	50.54
	−10 kPa	33.95	−0.166	85.64
50 °C	atm	35.02	−0.162	87.27
	−5 kPa	16.73	−0.108	51.55
	−10 kPa	33.87	−0.165	87.22
60 °C	atm	34.93	−0.162	88.81
	−5 kPa	16.65	−0.108	52.56
	−10 kPa	33.78	−0.165	88.80

### Economic analysis

The performance analysis of DAVD is a quantitative assessment of economic processes that may aid policymakers, investors, and food processors in making educated decisions on crop drying systems. The study employed Eqs. 24–35, wherein the lifetime reduction strategy integrates the basic recovery process. The analysis considered critical variables, as evidenced by the data in Tables 7 and 8, while also accounting for the condition of the Libyan economy and the projected costs related to the DAVD components. Table 7 demonstrates that the capital cost of the DAVD is about 300 USD, significantly lower than alternative drying systems, with a life expectancy of 20 years. Table 7 indicates that the yearly capital and investment costs were 20.16 USD and 19.16 USD, respectively.

**Table 7:** Various costs related to the DAVD for drying Sage leaves.

Cost parameters	Unit
Capital cost, USD	300
Lifespan, years	20
Annual capital cost, USD	20.16
Annual maintenance cost, USD	0.61
Annual salvage value, USD	1.61
Annualized investment cost, USD	19.16

In Table 8, the bold numbers in colored boxes represented the lowest payback period at different drying temperatures and an operating pressure. It can be concluded that the lowest payback period was 0.091 year (about 1.11 months), achieved at a drying temperature of 60 °C and operating pressure of – 10 kPa, where the drying time is the lowest (90 min) and the annually dried qualities of sage leaves are the highest compared to other drying temperatures and operating pressures.

**Table 8:** Economic parameters of the DAVD for drying Sage leaves.

Economic analysis	Drying temperature, °C & operating pressure, kPa								
	40 °C			50 °C			60 °C		
	atm	−5 kPa	−10 kPa	atm	−5 kPa	−10 kPa	atm	−5 kPa	−10 kPa
Specific energy consumption, KJ/kg	7344	5091.72	4095	5148	4728.4	3383.5	4006.8	3844.8	2160
Quantity of dried sage leaves, kg/year	823.6	1400	2000	1400	1750	2333.3	2000	2800	4666.7
Saving after 1 year	617.65	1050	1500	1050	1312.5	1750	1500	2100	3500
Payback period, year	0.509	0.301	**0.211**	0.301	0.241	**0.181**	0.211	0.151	**0.091**

## Conclusions, recommendations, and future work

In the present study, a Developed Automatic Vacuum Dryer (DAVD) was employed to investigate the drying kinetics of sage leaves under varying conditions. The experiments were conducted at three drying temperatures (40, 50, and 60 °C), three operating pressures (atmospheric pressure, − 5 kPa, and − 10 kPa), and a constant layer thickness of 1 cm. The key findings of this study are summarized as follows:

- Reducing the operating pressure from atmospheric pressure to − 10 kPa significantly decreased the drying time, with reductions of 142.9%, 100%, and 133.3% at drying temperatures of 40 °C, 50 °C, and 60 °C, respectively.
- The maximum effective moisture diffusivity (EMD) of 6.716 × 10⁻⁹ m²/s was achieved under optimal drying conditions at 60 °C and − 10 kPa, demonstrating the combined effect of elevated temperature and reduced pressure in significantly enhancing moisture migration.
- The minimum activation energy (19.4 kJ/mol) was achieved at an operating pressure of -10 kPa, indicating that higher vacuum levels significantly reduce the energy barrier for moisture diffusion.
- The drying behavior of sage leaves was analyzed using thin-layer drying models, with the Page model proving to be the most accurate in describing the drying characteristics. This model provided the best fit for the experimental data, confirming its suitability for predicting moisture loss in sage leaves under vacuum drying conditions.
- Thermodynamic parameters, including enthalpy (ΔH‡), entropy (ΔS‡), and Gibbs free energy (ΔG‡), were evaluated. The results (ΔH‡ > 0, ΔS‡ < 0, and ΔG‡ > 0) indicate that the drying process is endothermic (requiring energy input), Non-spontaneous (requires external conditions to proceed), and Entropically unfavorable (more ordered system during drying).
- An economic analysis of the DAVD revealed that drying sage leaves at 60 °C and − 10 kPa not only optimized drying efficiency but also reduced the payback period to less than two months, demonstrating its cost-effectiveness for industrial applications.

### Recommendations

The study highlights that vacuum drying at elevated temperatures (60 °C, − 10 kPa) significantly improves drying efficiency while maintaining economic feasibility. The Page model effectively describes the drying kinetics, and thermodynamic analysis confirms the energy-intensive nature of the process. These insights can be valuable for scaling up sage leaf drying operations in the food and pharmaceutical industries, where preservation of bioactive compounds and energy efficiency are critical.

### Future works

Future research should explore the effects of higher vacuum levels and varying layer thicknesses on drying efficiency and phytochemical retention in sage leaves. Advanced modeling (CFD, ANN) could optimize drying kinetics, while pilot-scale trials and hybrid drying systems (e.g., vacuum-microwave) should assess industrial scalability. Additionally, comparing vacuum drying with other methods (freeze-drying, convective drying) in terms of energy use and product quality is essential. Further studies could extend this approach to other herbs and heat-sensitive materials for broader applications.

## Data Availability

All data are provided within the article.
